# Supplementary material for: Tunable anomalous Hall conductivity through volume-wise magnetic competition in a topological kagome magnet
Source: Nat Commun. 2020 Jan 28;11:559. doi: 10.1038/s41467-020-14325-w (PMC6987130; doi:10.1038/s41467-020-14325-w)
Supplement: Supplementary file 1 — Supplementary Information [file 41467_2020_14325_MOESM1_ESM.pdf]

preprint(December 13, 2019)

Tunable Anomalous Hall Conductivity Through Volume-wise Magnetic Competition in a Topological Kagome Magnet $Co_3Sn_2S_2$

PACS numbers:

Supplementary Note 1: Temperature dependence of the structural parameters in $Co_3Sn_2S_2$

The crystal structure of $Co_3Sn_2S_2$ in the whole temperature range was well refined through Rietveld refinements to the raw neutron diffraction data, employing a rhombohedral lattice structure in the space group the R-3m. An example of the refinement profile for 200 K data is shown in Supplementary Fig. 1a. No secondary phase can be detected. Lattice constants a and c show a smooth temperature dependence, as depicted in Supplementary Fig. 1b. We observe a strong temperature dependence of the width of the diffraction peaks. The standard Cagliotti model of the peak broadening as a function of scattering angle contains two main contributions [1]: Gaussian broadening U proportional to the microstrain and Lorentzian broadening Y inversely proportional to the crystal domain size. As one can see from the Supplementary Fig. 1c and d, both parameters increase below the transition temperature. This effect is difficult to rationalize without a phase transition. In the present case, it seems that there is substantial coupling between the magnetic ordering and the crystal lattice, so that the ordered magnetic domains effectively shrink the sizes of coherently scattering regions and create the microstrains on the temperature lowering.

Supplementary Note 2: Field effect on the temperature dependence of the muon spin relaxation rate in $Co_3Sn_2S_2$

Supplementary Fig. 2a shows the temperature dependence of the transverse relaxation rates λ_{T1} and λ_{T2} for $Co_3Sn_2S_2$ for the high frequency and the low frequency components, respectively, taken in zero magnetic field. The temperature dependence of λ_{T2} is also shown under various applied magnetic fields up to $\mu H_{\rm ext}$ = 8 T. In zero field, λ_{T1} is nearly constant and remains relatively low up to the transition temperature $T_{\rm c2}$ and then becomes zero. The low frequency signal always exhibits a higher relaxation rate compared to the high frequency component. Moreover, λ_{T2} increases with increasing the temperature and shows the maximum near the transition temperature $T_{\rm c1}$ with the much higher value of λ_{T2} = 8.9(3) μs^{-1} as compared to the one at 90 K. This implies that the second magnetic component becomes more disordered upon approaching the transition. Upon applying the magnetic field, the peak vanishes at a field of 0.1 T and a monotonous increase of the relaxation rate is observed upon lowering the temperature, which is typical for the field induced polarised state. Furthermore, the low temperature value of the relaxation rate saturates for $\mu H_{\rm ext} > 1$ T, indicating that the sample is in a fully polarised FM state. These results imply that relatively low fields are enough to tune the magnetic state in $Co_3Sn_2S_2$, which is consistent with the magnetization data, shown below.

Supplementary Note 3: Probing the Macroscopic Magnetic Properties in $Co_3Sn_2S_2$

μSR data, presented in the main text clearly demonstrate the presence of novel magnetic transition/crossover in $Co_3Sn_2S_2$ in addition to the magnetic transition at $T_{\rm C}$ = 177 K, known from the literature. In order to substantiate the presence of the novel magnetic phase transition in $Co_3Sn_2S_2$, we carried out temperature and field dependent magnetization experiments. The temperature dependence of the zero-field-cooled (ZFC) (sample was cooled down to the base-T in zero magnetic field and the measurements were done upon warming) and field-cooled (FC) (the sample was cooled down to the base-T in an applied magnetic field and the measurements were done upon warming) macroscopic magnetic moment for $Co_3Sn_2S_2$, recorded in low field of $\mu_0 H = 1$ mT and under various applied fields up to 6 T, are shown in Supplementary Figs. 2b and c, respectively. The sharp increase of the moment with decreasing temperature in the temperature range $T_{\rm C1} \simeq 177$ K to $T_{\rm C2} \simeq 172$ K, along with the same value for both ZFC and FC moment, indicates the fully polarized FM state. The difference between the ZFC and FC response is seen below 172 K, indicating the formation of some ferromagnetic domains. With decreasing temperature from 172 K, the ZFC moment decreases rapidly, but below 140 K, the decrease is less pronounced. Instead, the moment increases and shows a broad maximum at $T_{\rm C}^{*} \simeq 120$ K. A similar behaviour is observed under an applied magnetic fields, but the anomaly shifts towards lower temperatures. Above 60 mT, the fully polarized ferromagnetic state is stabilised. Note that within the classical picture one would expect the monotonous decrease of the moment below $T_{\rm C}$. Thus, the observed anomaly points to some novel magnetic transition within the magnetically ordered state. The SQUID data are consistent with the μSR results and can be considered as additional independent piece of evidence for the complex temperature evolution of the magnetic state in $Co_3Sn_2S_2$. Based on these data, we can construct a temperature-

field phase diagram for $Co_3Sn_2S_2$. Shown in Supplementary Fig. 2d are the field dependences of the following temperatures, T_{C1}, T_{C2} and T_C^*, marked by the arrows in Supplementary Fig. 2b and c. Four different magnetic phases were identified: a paramagnetic (PM), FM with moments along c-axis and AFM within the plane, and a fully polarised ferromagnetic phase.

Supplementary Note 4: Pressure-dependent magnetic order in $Co_3Sn_2S_2$

For further insight into the magnetic order in $Co_3Sn_2S_2$, ZF μSR experiments were carried as a function of hydrostatic pressure. The temperature dependences of the internal field $\mu_0 H_{int}$, magnetically ordered volume fraction and the relaxation rates λ_1 and λ_2 for $Co_3Sn_2S_2$, recorded at various applied pressures, are shown in Supplementary Figs. 3a-d. We find that hydrostatic pressure has a significant effect on the magnetic properties of these materials. Namely, the pressure causes a substantial reduction of $\mu_0 H_{int}$, T_{C1}, T_{C2} and T_C^* while keeping the fully magnetically ordered volume fraction intact. The fact that under pressure the in-plane AFM component appears at lower temperature as compared to the one at ambient pressure, implies that the pressure tends to stabilize the high temperature in-plane AFM structure. The Strong pressure dependence of magnetism is very encouraging, since it implies that one can have control over the magnetic properties in this topologically non-trivial material. Currently, we do not yet adequately understand this behavior to comment further. Further experimental and theoretical efforts are certainly needed for understanding pressure dependent data.

Supplementary Note 5: Magnetic domains and Hysteresis

It is interesting to check with μSR how the magnetic response of two magnetically ordered regions in $Co_3Sn_2S_2$ changes after initially applying a high enough magnetic field to get the fully polarised state and the subsequent removal of the magnetic field. For this purpose, we did the comparison between the measurements, carried out in zero-field cooled and field cooled mode at the fixed temperature. The measurements were done at 170 K, where two clearly separated peaks, arising from two different magnetically ordered regions are observed. Namely, the following measurements were carried out:
(a) Zero-field cooling: The sample was cooled down from the paramagnetic state in zero-field to 5 K, then warmed up to 170 K and the spectrum was recorded. (b) Field cooling in an applied magnetic fields of 400 mT and 750 mT: The sample was warmed up to 200 K, where the sample is in the paramagnetic state. Then, the magnetic field was applied. To keep the same temperature cycling history, the sample was first cooled down to 5 K in the applied magnetic field, then warmed up to 170 K. Field was set to zero and the spectrum was recorded again in zero applied field.

Supplementary Fig. 4a shows the Fourier transform amplitudes of the oscillating components of the μSR time spectra, recorded at 170 K after zero-field cooled and field cooled conditions. The narrow and the broad components arise from the magnetically ordered regions with the out-of plane FM structure and in-plane AFM structure, respectively. A systematic reduction of the fraction of the narrow component is observed (see Supplementary Fig. 4b) as a result of the above mentioned field cycling experiment. This implies that the domains do not fully return to their original state and there is some redistribution of the magnetically ordered regions confirming the phase separation scenario.

Supplementary Note 6: Muon site estimation for $Co_3Sn_2S_3$ using DFT

DFT calculations were performed using plane waves as a basis set, using the computational implementation in the form of Quantum Espresso package [2]. The crystal structure for the computation was taken from FIZ Karlsruhe Inorganic Crystal Structure Database. The atoms were represented using ultrasoft pseudopotentials from GBRV library [3]. The exchange and correlation were described using the Generalized Gradient Approximation of the Perdew-Burke-Ernzerhof flavor [4]. The plane wave expansion was carried out up to a kinetic energy cut-off of 200 Ry and up to 1500 Ry for the charge density. The reciprocal space was sampled using an 4x4x4 Monkhorst-Pack grid [5].

After the electron density of the material was obtained self-consistently, the electrostatic potential was calculated within the unit cell. The muon site is assigned to the position with a minimum electrostatic potential. Within the unit cell, the minima of the electrostatic potential are found at the crystallographic Wyckoff position 36i (see Supplementary Fig. 5). No other local minima were found within any reasonable energy range and the different 36i positions are separated by a potential barrier of the order of 0.1 eV, which suggests that the muons are locked into the sites after thermalization.

Supplementary Note 7: Local field calculations

Internal field calculations at the single 36i muon site were performed using the Python package *Muesr* [6]. A sphere with a radius large enough to encapsulate 50 unit cells was constructed and dipole summation over all mo-

ments in the sphere was performed to find the local field at the muon site. Two magnetic models were calculated: the out-of-plane ferromagnetic $R-3m'$, and the in-plane antiferromagnetic $R-3m$ structures. A model featuring a weighted sum of each magnetic structure was fitted to the experimental time-spectra using a Python implementation of the *Minuit2* fitting algorithm [7], with the moment size, volume fraction and the relaxation rates as model parameters. The temperature dependences of the moment size, the volume fractions of the FM and AFM components as well as the transverse and the longitudinal relaxation rates are shown in Supplementary Figs. 6a-d. The resulting fits yield a physical interpretation of the two-component signal observed in zero-field μSR, and the magnitude of the moments are in good agreement with the less sensitive estimations from neutron diffraction.

Supplementary Note 8: Velocity of the Fermi arc in $Co_3Sn_2S_2$

Supplementary Figs. 7a-d show the raw ARPES spectrum, representative momentum distribution curve at the Fermi level, first-principles spectrum along the loop and unsymmetrized ARPES spectrum, respectively. A fit to the train of peaks (white line) yields a Fermi velocity of $v = 0.62 \pm 0.05$eV Å for the Fermi arc. Supplementary Fig. 7c shows a Fermi arc connecting the valence and conduction bands.

Supplementary Note 9: Temperature evolution of the anomalous Hall conductivity and the ordered moment

We demonstrate in the main paper that the temperature evolution of anomalous Hall conductivity (AHC) follows extremely well with the evolution of the ordered volume fraction, i.e., the magnitude of AHC is robust against the increased temperature up to 90 K and it gradually decreases above $T \sim 90$ K, which corresponds to the temperature above which the fraction of the in-plane AFM structure increases. Here, we compare the temperature dependence of the anomalous Hall conductivity to the temperature evolution of the the order parameter (see Supplementary Fig. 8), recorded in zero-field and in the applied field of 8 T. It is clear that the AHC of $Co_3Sn_2S_2$ has different temperature evolution than the ordered moment size. This indicates that the magnitude of AHC does not depend on the magnetic moment size, but it‘s temperature evolution is rather determined by the magnetically ordered fraction.

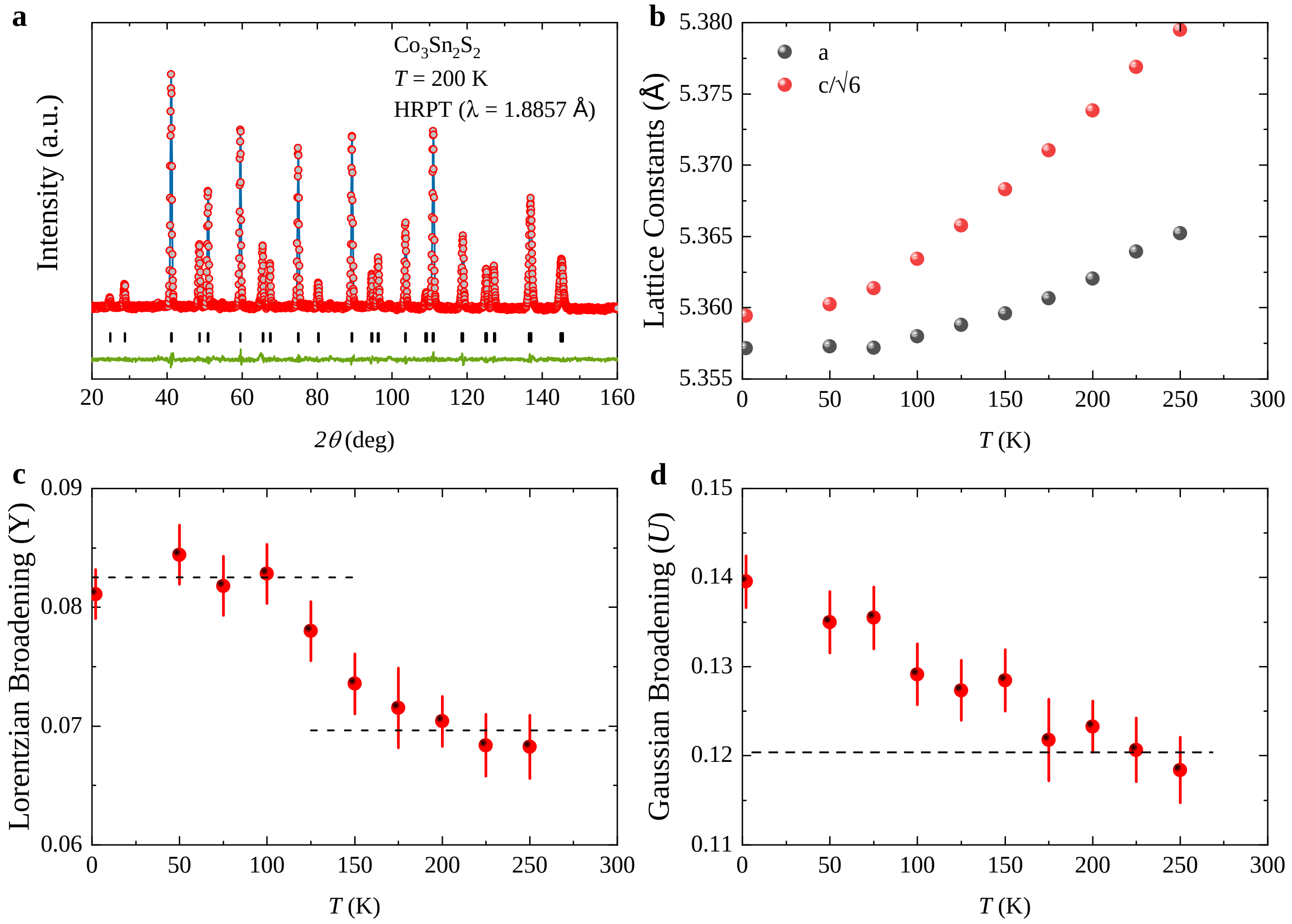

Supplementary Fig. 1: **The temperature dependent lattice parameters of $Co_3Sn_2S_2$.** Neutron powder diffraction pattern, recorded at 200 K (a) for the sample $Co_3Sn_2S_2$. The solid blue line represent a Rietveld refinement profile. The residuals are plotted at the bottom of the figure. The temperature dependence of the lattice constants (b), the Lorentzian broadening (c) and the Gaussian broadening (d).

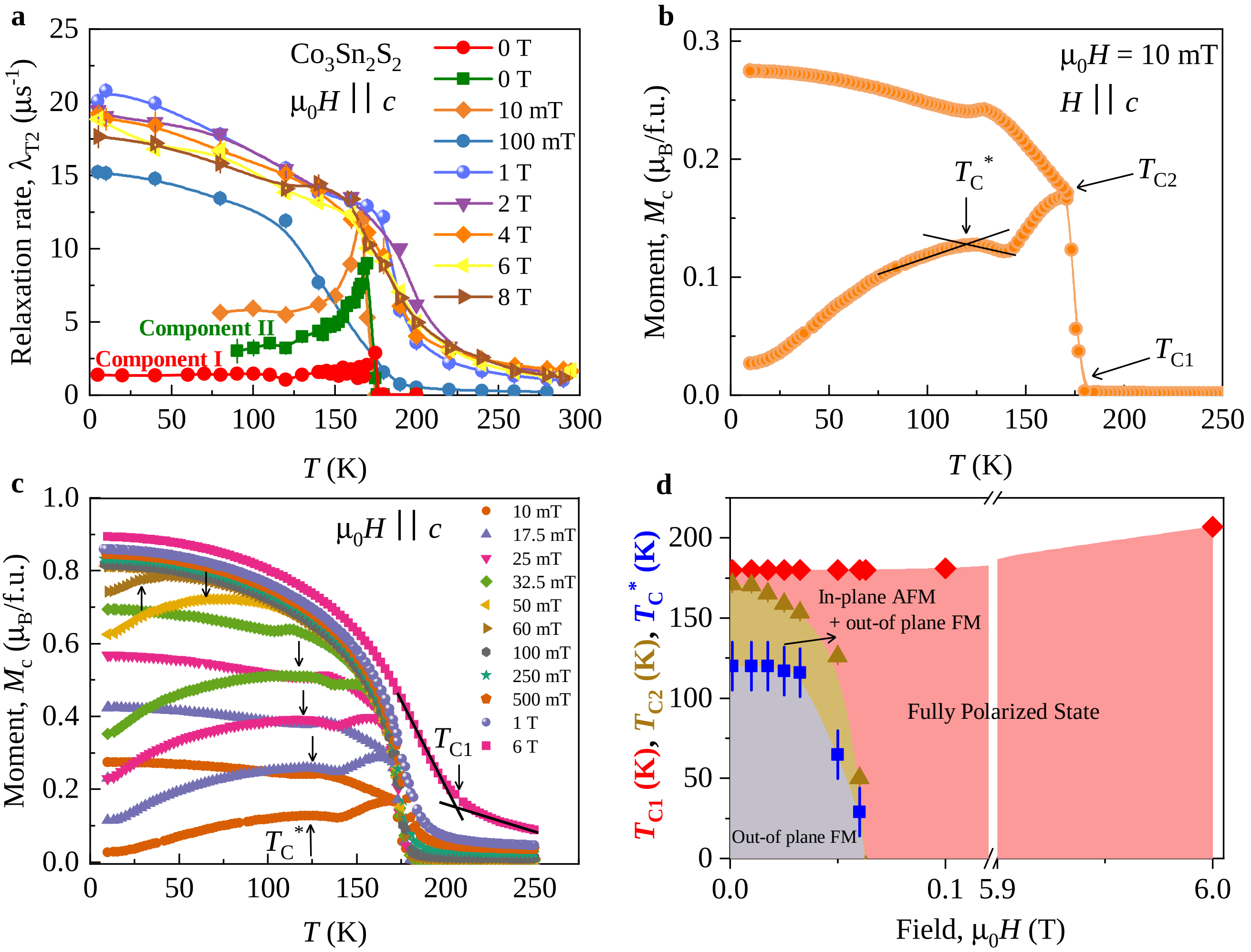

Supplementary Fig. 2: **Temperature and field dependence of the bulk magnetization and the muon-spin relaxation rate for $Co_3Sn_2S_2$.** (a) The temperature dependence of the muon-spin relaxation rate, measured at various fields up to 8 T. The temperature dependence of the magnetic moment, measured in an applied field of 1 mT (b) and at higher fields up to 6 T (c). (d) Temperature-Field phase diagram for $Co_3Sn_2S_2$.

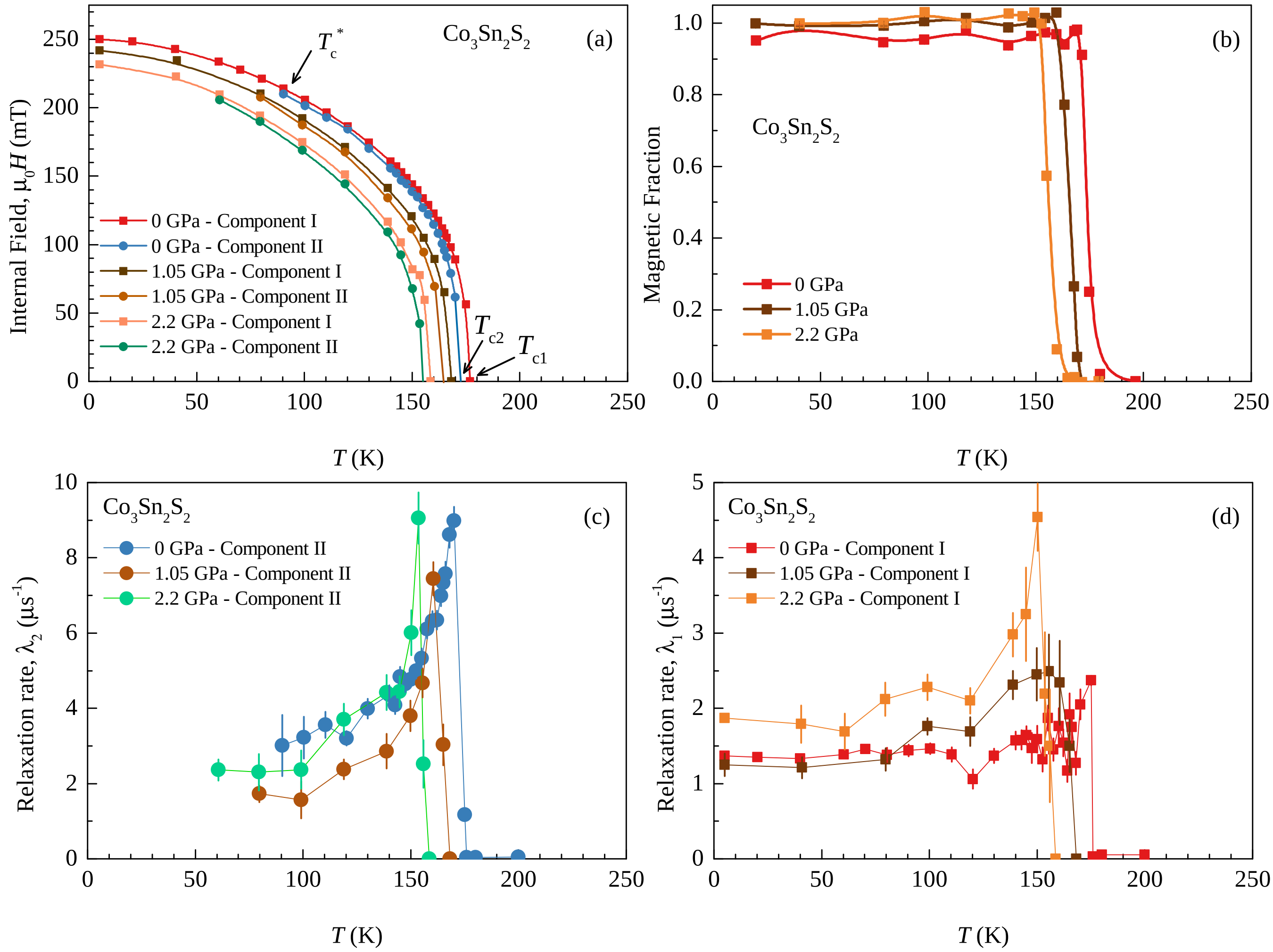

Supplementary Fig. 3: **Pressure effect on magnetic quantities in $CoSn_2S_2$.** (a) The temperature dependences of the internal magnetic fields (a), the magnetic volume fraction (b), and the transverse relaxation rates for the low frequency (c) and high frequency components (d), measured at various pressures up to 2 GPa. Arrows mark the transition temperatures T_{C1}, T_{C2} and T^*_C.

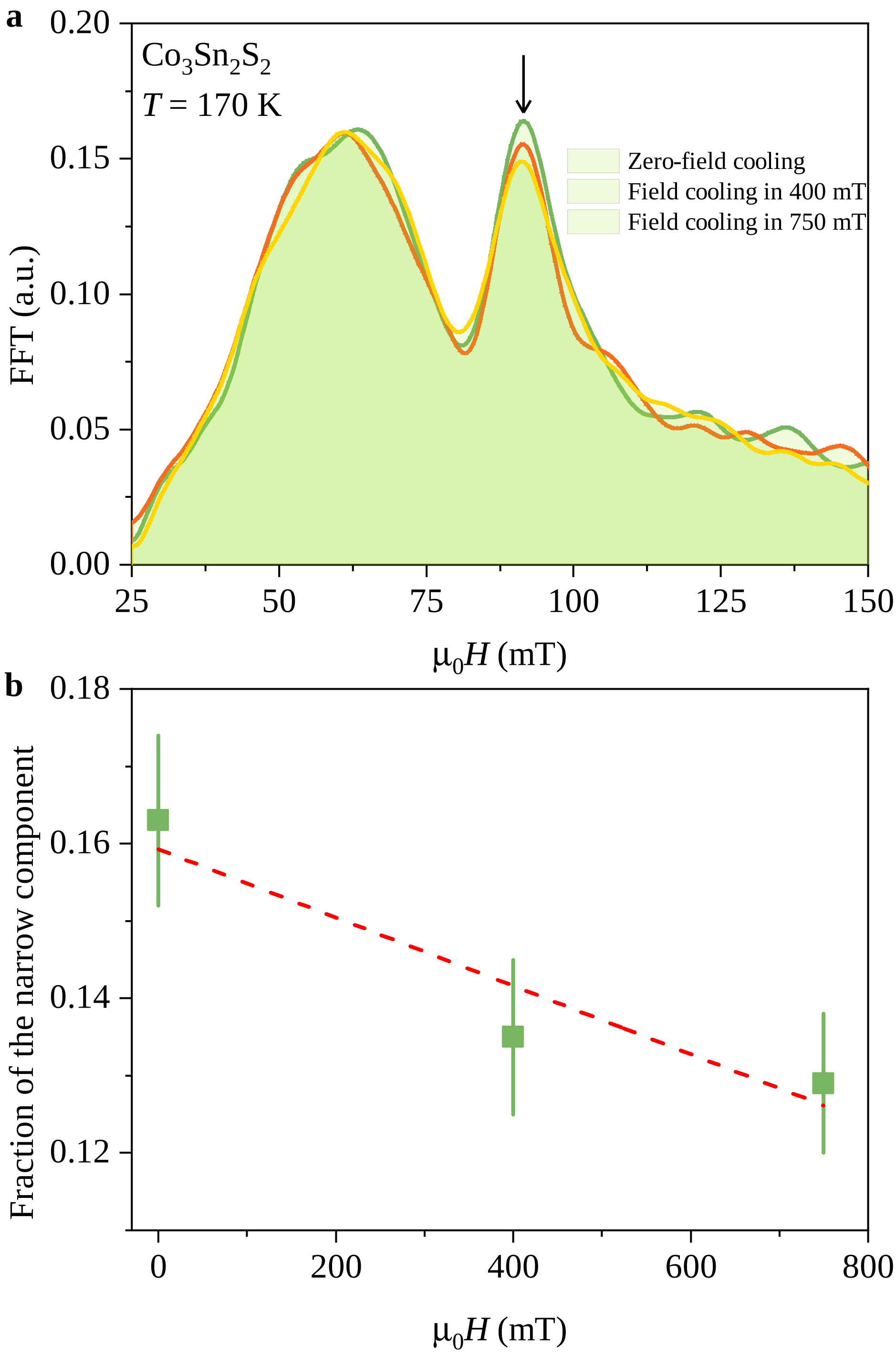

Supplementary Fig. 4: **Magnetic hysteresis in $Co_3Sn_2S_2$.** (a) Fourier transform amplitudes of the oscillating components of the μSR time spectra for $Co_3Sn_2S_2$, recorded at 170 K after zero-field cooled and field cooled conditions. (b) The field dependence of the relative volume fraction of the ferromagnetically ordered region.

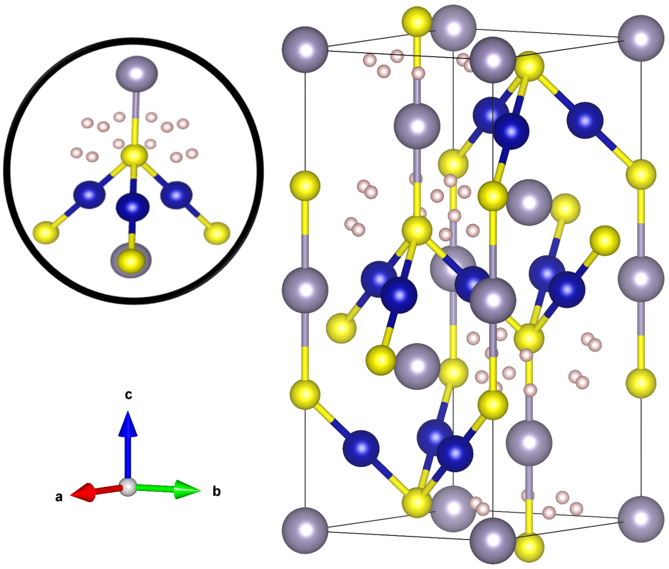

Supplementary Fig. 5: **Muon stopping sites in $Co_3Sn_2S_2$.** Crystalographically equivalent muon stopping sites with the Wyckoff position 36i within the structure of $Co_3Sn_2S_2$.

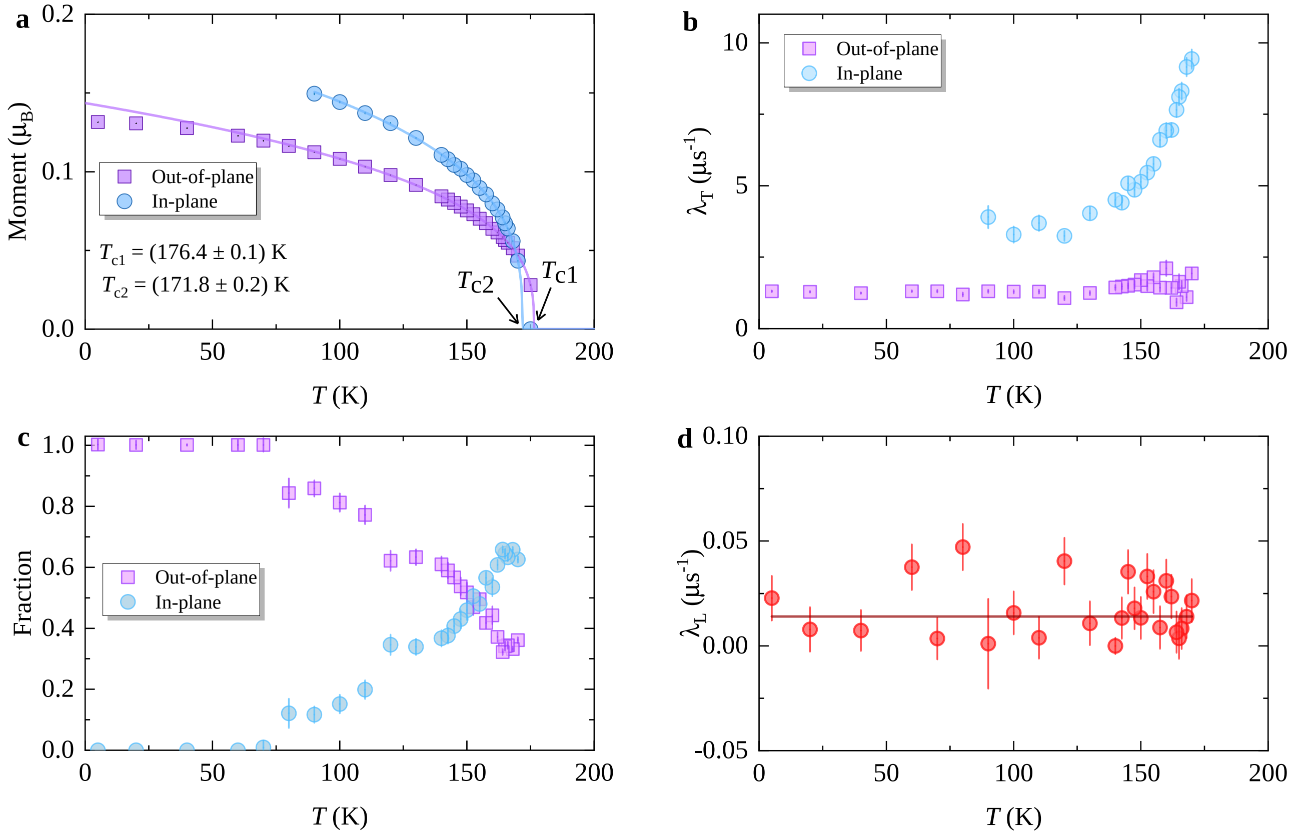

Supplementary Fig. 6: **Results of the local field simulation in $Co_3Sn_2S_2$.** The temperature dependence of the ordered moments (a), the transverse depolarization rates (b), and the relative fractions (c) for $Co_3Sn_2S_2$ for the out-of-plane ferromagnetic and in-plane antiferromagnetic configurations. (d) Longitudinal depolarization rate as a function of temperature for $Co_3Sn_2S_2$.

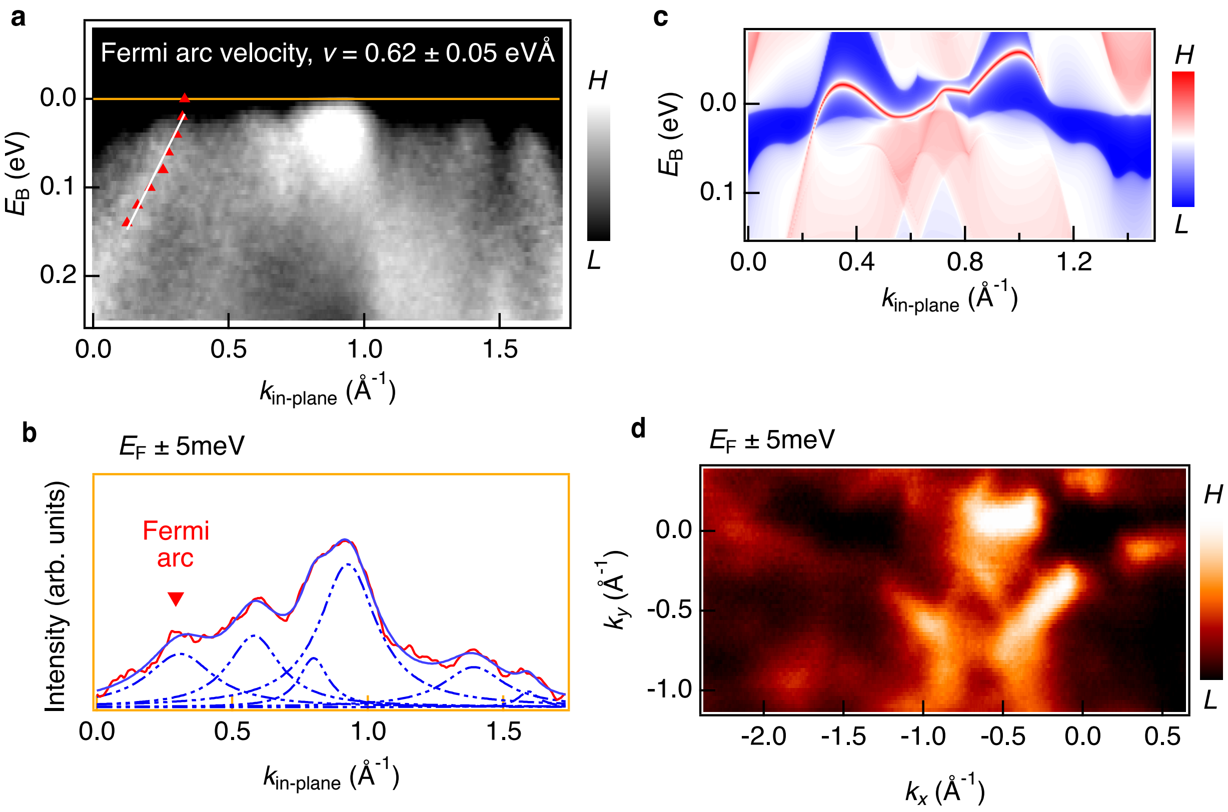

Supplementary Fig. 7: **Velocity of the Fermi arc in $Co_3Sn_2S_2$.** (a) Raw ARPES spectrum used to produce main text Fig. 1d, with Fermi arc tracked (red triangles) using Lorentzian fits to momentum distribution curves (MDCs). A fit to the train of peaks (white line) yields a Fermi velocity of $v = 0.62 \pm 0.05$eV Å for the Fermi arc. (b) Representative MDC at the Fermi level (red curve) with fitted Lorentzian peaks (blue dotted curves) and their sum (blue solid curve). (c) First-principles spectrum along the loop indicated in main text Fig. 1e, clearly exhibiting a Fermi arc connecting the valence and conduction bands. (d) Unsymmetrized ARPES spectrum used for main text Fig. 1c.

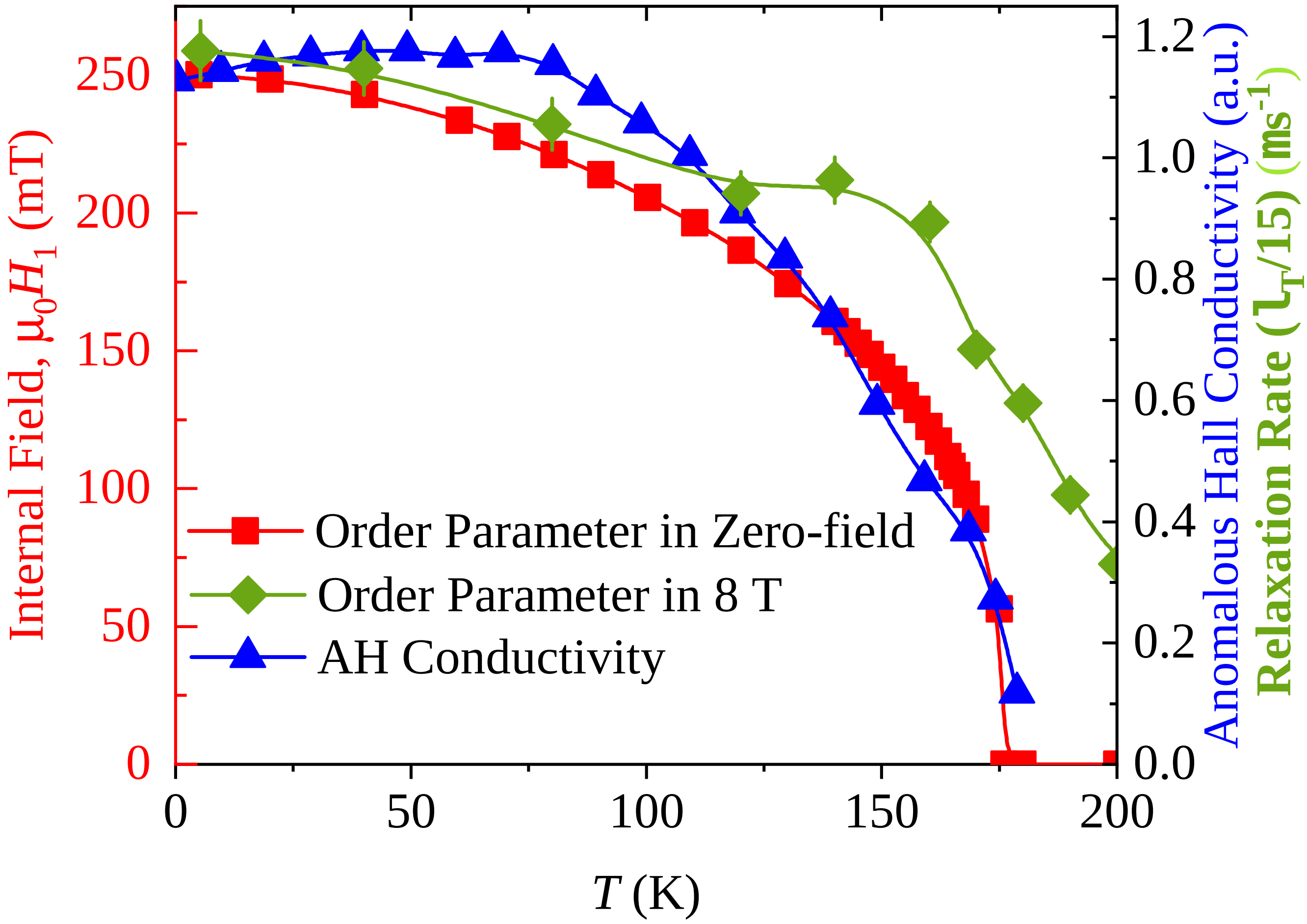

Supplementary Fig. 8: **The magnetic order parameter and AHC for $Co_3Sn_2S_2$.** The temperature dependence of the internal field, the relaxation rate, measured in the highest applied field of 8 T and the anomalous Hall conductivity for $Co_3Sn_2S_2$.

Supplementary References

[1] Rodriguez-Carvajal, J. Phys. B **192**, 55 (1993).
[2] Giannozzi et al., JPCM**21**, 395502 (2009).
[3] Garrity et al., CMS **81**, 446 (2014).
[4] Perdew et al., PRL **77**, 3865 (1996).
[5] Monkhorst and Pack, PRB **13**, 5188 (1976).
[6] Pietro Bonfá, Ifeanyi John Onuorah, and Roberto De Renzi, JPS Conf. Proc. **21**, 011052 (2018)
[7] M. Hatlo et. al., IEEE Trans. Nucl. Sci. **52**, (2005).
